# Integrated NSM and GFRP-reinforced ECC/UHPC techniques for strengthening deficient RC columns

**DOI:** 10.1038/s41598-026-52870-4

**Published:** 2026-05-27

**Authors:** Galal Elsamak, Alireza Bahrami, Mohamed Emara, Yahya M. Bin Mahfouz, Yahia Iskander, Mohamed Ghalla

**Affiliations:** 1 Civil Engineering Department, Faculty of Engineering, Kafrelsheikh University, Kafrelsheikh, Egypt; 2 Department of Building Engineering, Energy Systems and Sustainability Science, Faculty of Engineering and Sustainable Development, University of Gävle, 801 76 Gävle, Sweden; 3 Structural Engineering Department, Faculty of Engineering, Zagazig University, Zagazig, 44519 Egypt; 4 Civil Engineering Department, College of Engineering and Architecture, Umm Al-Qura University, Makkah, Saudi Arabia; 5 Faculty of Engineering and Digital Technologies, University of Bradford, Bradford, BD71DP UK

**Keywords:** Column, NSM, Glass fiber-reinforced polymer, Engineered cementitious composite, Ultra-high-performance concrete, Strengthening, Engineering, Materials science

## Abstract

The integrity of the structure and load capacities of reinforced concrete (RC) columns are severely impacted by corrosion-induced reinforcing steel losses. This study examines an innovative strengthening technique incorporating near-surface mounted (NSM) steel or glass fiber-reinforced polymer (GFRP) bars, combined with an external layer of engineered cementitious composite (ECC) or ultra-high-performance concrete (UHPC) reinforced with a GFRP mesh. The experimental results demonstrated that UHPC provided superior confinement compared with ECC jackets, leading to the highest load capacity enhancement. Incorporating NSM steel bars with ECC (Group 3 (G3)) significantly improved performance, increasing ultimate load capacity by 25%–32%, with energy absorption increasing up to 3.9 times compared with the control column. The combination of NSM bars with external jacketing proved to be the most effective, particularly when NSM GFRP bars were used alongside UHPC, resulting in a 49% increase in load capacity and a substantial improvement in energy absorption. Additionally, finite element models were developed and showed good agreement with the experimental findings, further validating the proposed strengthening technique. The model was extended for a parametric study examining the effect of main reinforcement diameter. The results highlight the important role of the reinforcement diameter in enhancing column strength. Increasing the reinforcement diameter led to progressive strength improvements, but the efficiency of additional reinforcement decreased at larger diameters.

## Introduction

Reinforced concrete (RC) buildings are often exposed to different loading regimes and destructive environmental factors, leading to deterioration over time ^1–5^. Among the primary causes of degradation, reinforcement corrosion significantly affects the structural integrity of RC members. This deterioration occurs through multiple mechanisms, including the reduction of the steel cross-sectional area, the formation of expansive corrosion products that induce cracking and spalling of the concrete cover, and the weakening of the steel-to-concrete bond ^6–9^. In RC columns, which are crucial for resisting axial compressive loads, corrosion-induced damage severely compromises performance ^10,11^. The loss of strength and ductility due to deficient reinforcement, combined with concrete cracking and section loss, leads to reduced load capacity and stiffness ^12,13^. Furthermore, corrosion of transverse stirrups weakens concrete confinement, further diminishing the column’s ductility and overall structural stability. As a result, the axial load capacity, stiffness, and deformation capacity of deficient RC columns are considerably impaired, increasing the risk of premature failure ^14^.

Nowadays, high-performance concretes (HPCs), such as ultra-high-performance concrete (UHPC) and engineered cementitious composites (ECCs), have gained widespread use due to their superior properties compared with normal concrete (NC), particularly in terms of enhanced tensile strength and exceptional durability under harsh environmental conditions. Their advanced mechanical performance and resistance to aggressive factors make them preferred choices for modern construction applications requiring longevity and resilience ^15–17^. The strengthening of RC columns using ECC and UHPC has gained attention due to their superior mechanical properties and durability. Both materials improve the load capacity, stiffness, and ductility of existing columns, making them suitable for various structural applications ^18–20^.

ECC jackets can enhance the load capacity by up to 2.5 times compared with control columns. Moreover, the energy absorption capacity of ECC-strengthened columns is significantly improved, in initial and secant stiffness values increasing by up to 3.2 times. Additionally, effective surface preparation techniques, such as longitudinal grooving, are crucial for maximizing the bond between ECC and the existing concrete ^21^.

UHPC jackets improve the axial load capacity and moment capacity of RC columns, with the effectiveness being proportional to the jacket thickness ^22–24^. UHPC is particularly effective under eccentric loading conditions, demonstrating improved toughness and moment capacity. The integration of steel bars within UHPC jackets leads to notable enhancements in stiffness and load capacity ^25,26^. In contrast, while both ECC and UHPC offer substantial benefits for column strengthening, the choice between them may depend on specific project requirements, such as environmental conditions and load scenarios. Each material presents unique advantages that can be leveraged for optimal structural performance. The integration of UHPC/ECC jackets reinforced with steel/FRP mesh presents a promising solution for enhancing the seismic resilience of existing structures. This innovative approach not only rehabilitates damaged joints but also significantly improves their performance under seismic loads. This technique helps enhance the compressive strength and ductility of RC columns. It provides strong confinement, delays crack development, and improves mechanical performance ^27^. Compared with unreinforced columns, mesh-reinforced ECC layers increase cracking load by about 107.3% and peak capacity by about 104%, while also outperforming ECC-reinforced columns. Ductility improves by 75.6%–77.8% over unreinforced columns and by 17.3%–18.6% over ECC-reinforced ones ^28^. UHPC jackets reinforced with high-strength steel mesh can increase the peak strength by up to 93%. The use of these jackets effectively prevents joint shear failure, allowing for improved flexural behavior during cyclic loading. UHPC jackets enhance energy dissipation by 140% and initial stiffness by approximately 35% ^29,30^.

The near-surface mounted (NSM) technique for enhancing RC columns has gained popularity owing to its effectiveness in improving load capacity and ductility. This method involves embedding reinforcement bars, such as steel or fiber-reinforced polymers (FRPs), into grooves on the column surface, which can considerably improve structural performance under various loading conditions. Combining NSM steel bars with glass FRPs (GFRPs) or carbon FRPs (CFRPs) laminates has shown substantial improvements in load capacity. For instance, hybrid NSM systems can increase the ultimate load by up to 80% compared with unstrengthened columns ^31^. Under eccentric loading, columns strengthened with NSM techniques exhibited increases in ultimate load capacity of 62% to 79.45% compared with control specimens ^32^.

Various studies have validated the effectiveness of NSM strengthening through both numerical simulations and experimental tests, demonstrating good agreement in load–displacement behavior. Moreover, research indicates that increasing the number of NSM bars improves the load capacity, while factors like the slenderness ratio can negatively impact performance ^33,34^.

The use of NSM reinforcement has been linked to improved ductility, particularly when combined with FRP jackets, which can enhance shear capacity and prevent buckling ^35^.

The use of longitudinal NSM CFRP laminates considerably enhanced the load capacity of concrete columns, enabling them to withstand peak eccentric loads without crushing, debonding, or local buckling. The study assessed performance under varying eccentricities (0%, 10%, 20%, and 30% of the specimen width) and demonstrated that NSM CFRP laminates effectively improved the compressive strength of the tested columns ^36^.

Abdulghafoor and Al-Baghdadi ^37^ investigated the NSM CFRP strengthening technique, which, when applied to RC columns, resulted in a significant increase in load capacity of approximately 46.6% compared with unstrengthened columns. The NSM CFRP technique was tested alongside other methods, including hybrid CFRP and externally bonded CFRP, under both static and dynamic conditions. The findings highlighted the effectiveness of NSM CFRP in enhancing the seismic performance of existing RC slender columns under lateral loads.

While NSM strengthening techniques show promising results, some studies suggest that the effectiveness can vary based on specific design parameters and loading conditions, indicating the need for tailored approaches in practical applications.

Although previous studies have examined NSM reinforcement or cementitious jacketing techniques separately, limited research has addressed their integrated application for strengthening deficient circular RC columns under axial loading. This study investigates a combined strengthening system using NSM steel or GFRP bars with ECC or UHPC jacketing reinforced by GFRP mesh, aiming to evaluate the interaction between these techniques and their effectiveness in improving the axial capacity and energy absorption of deficient columns. Most existing studies focus either on NSM reinforcement or externally bonded composites separately, lacking a comprehensive evaluation of their combined effect on structural performance, durability, and failure mechanisms. The interaction between NSM reinforcement and the external ECC/UHPC layer, particularly in terms of bond behavior, stress distribution, and long-term durability under aggressive environments, is not well understood. Additionally, limited studies explore the effectiveness of GFRP reinforcement in mitigating progressive deterioration, improving seismic performance, and ensuring enhanced ductility in deficient columns. Addressing these gaps through experimental and numerical investigations can lead to more efficient and durable strengthening solutions for deficient RC columns.

## Experimental investigation

### Experimental setup and column testing matrix

**Fig. 1:**
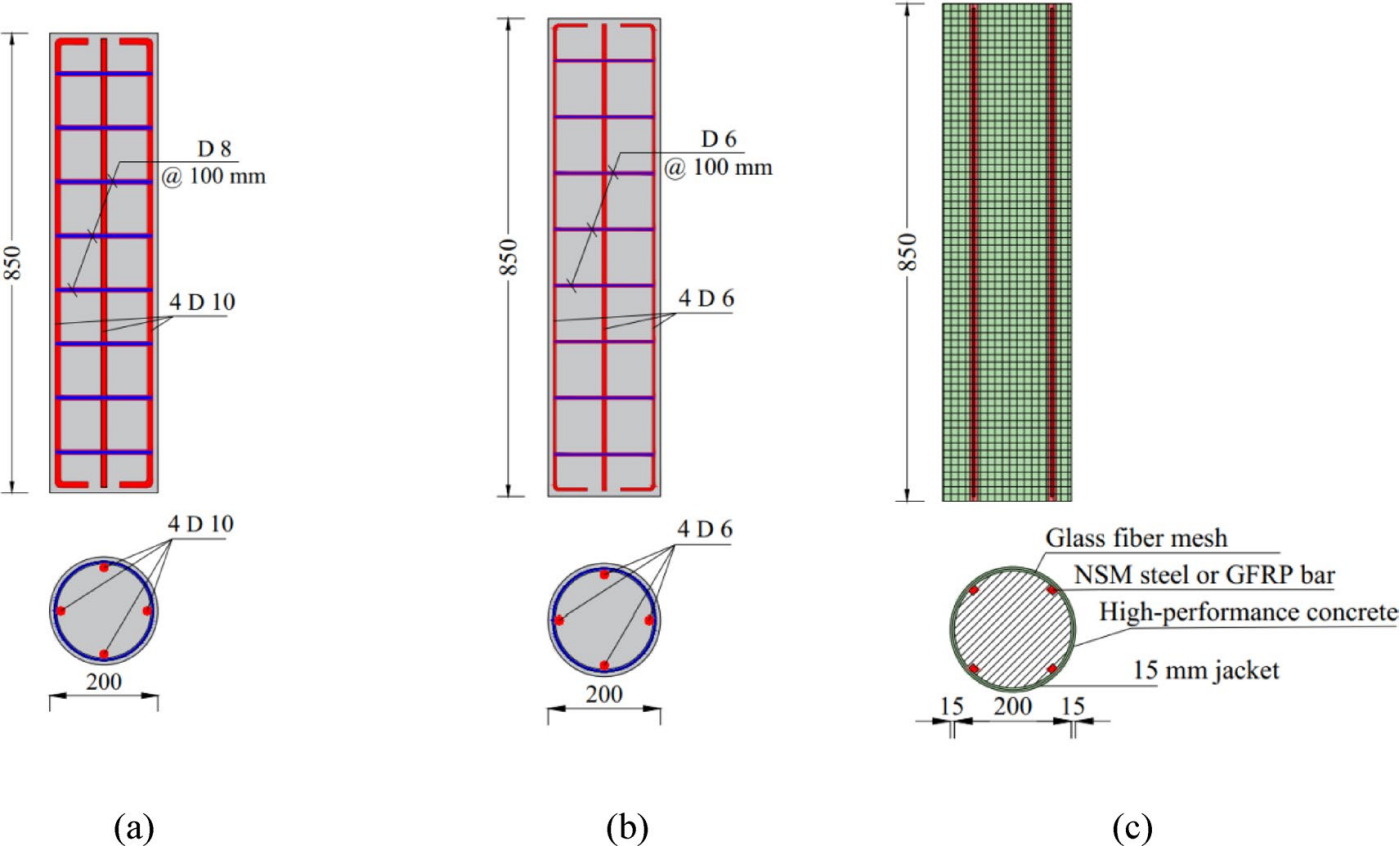
Geometric and reinforcement details of tested circular columns: (**a**) master column, (**b**) deficient column, and (**c**) strengthened column. (Units: mm).

Eleven short circular RC columns were tested under axial compression. Each column had a uniform cross-section with a diameter of 200 mm and a height of 850 mm, as illustrated in Fig. 1. According to Table 1, columns were classified into five different groups to investigate the effect of different parameters on the behavior of tested columns. The specimen notation used in this study describes the strengthening configuration applied to each column. The letter C refers to the control deficient column, while MC denotes the master column without reinforcement reduction. The symbols S and G indicate the use of NSM steel bars and NSM GFRP bars, respectively. The letters E and U represent the type of external jacket, namely ECC and UHPC, while the numbers 1 and 2 denote the number of GFRP mesh layers embedded within the jacket. This naming system facilitates the identification of the strengthening parameters for each specimen. The first group served as the control group. It contained two columns; one served as the master non-deficient column noted as MC (Fig. 1a), while the other specimen (Fig. 1b) served as the deficient column that experienced a reduction in reinforcement ratio (by reducing the bar diameter). The second group intended to investigate the effect of the quantity of GFRP meshes in the confining jacket, in which one and two GFRP layers were utilized for columns C-1-E and C-2-E, respectively. In order to examine the effect of utilizing the NSM technique to improve the behavior of confined RC columns, the third group was designed containing two columns strengthened with NSM steel bars and confined using an ECC layer. In one column (C-S-1-E), one layer of GFRP mesh was embedded in ECC, while two GFRP layers were used in the other column (C-S-2-E). The fourth group was similar to the third, with the primary distinction being the use of a UHPC jacket instead of the ECC jacket employed in the previous group. The final group comprised three specimens designed to assess the effect of incorporating four NSM GFRP bars. In one specimen (C-G-1-E), the bars were confined using an ECC layer reinforced with a single GFRP mesh. Another specimen (C-G-2-E) featured two GFRP mesh layers within the ECC confining layer. In the last specimen of this group (C-G-1-U), the GFRP bars were confined using a UHPC layer that included a single GFRP mesh layer.

**Table 1:** Test specimens.

Group	Column	GFRP layers	Jacket	Parameter
G1	MC	–	–	Control
	C	–	–	
G2	C	–	–	Number of GFRP layers
	C-1-E	One	ECC	
	C-2-E	Two	ECC	
G3	C	–	–	Existence of NSM steel bars + ECC jacket
	C-S-1-E	One	ECC	
	C-S-2-E	Two	ECC	
G4	C	–	–	Existence of NSM steel bars + UHPC jacket
	C-S-1-U	One	UHPC	
	C-S-2-U	Two	UHPC	
G5	C	–	–	Existence of NSM GFRP bars + ECC/UHPC jacket
	C-G-1-E	One	ECC	
	C-G-2-E	Two	ECC	
	C-G-1-U	One	UHPC	

MC: master column; C: deficient column; S: NSM steel bars; G: NSM GFRP bars; E: ECC; U: UHPC; 1: one layer of GFRP mesh; 2: two layers of GFRP mesh.

### Materials

Table 2 presents the mix proportions and compressive strengths of three different types of concrete: NC, ECC, and UHPC. With a water-to-binder ratio of 0.42, NC has a compressive strength of 30 MPa and is composed of 350 kg/m^3^ of cement, 700 kg/m^3^ of fine aggregate, and 1150 kg/m^3^ of coarse aggregate. In contrast, ECC contains 560 kg/m^3^ of cement, 442 kg/m^3^ of fine aggregate, 610 kg/m^3^ of fly ash, and 235 kg/m^3^ of silica fume, with a lower water-to-binder ratio of 0.25. Additionally, it incorporates 2.00% of PVA fibers by volume and 14.6 kg/m^3^ of high-range water reducer (HRWR), resulting in a significantly higher compressive strength of 62 MPa. UHPC exhibits the highest strength, reaching 126 MPa, and is characterized by a high cement content of 900 kg/m^3^, 1005 kg/m^3^ of fine aggregate, and 220 kg/m^3^ of silica fume. It has a water-to-binder ratio of 0.22, includes 2.00% of steel fibers by volume, and requires a substantially higher HRWR dosage of 40.3 kg/m^3^. The variations in mix proportions illustrate the material enhancements that contribute to the superior mechanical properties of ECC and UHPC compared with conventional NC.

**Table 2:** Mixture and strengths of concrete.

Concrete	Cement (kg/m^3^)	Fine aggregate (kg/m^3^)	Coarse aggregate (kg/m^3^)	Fly ash (kg/m^3^)	Silica fume (kg/m^3^)	Water/binder	Fiber (%) in volume	HRWR (kg/m^3^)	f’_*c*_ (MPa)	f_t_ (MPa)
NC	350	700	1150	–	–	0.42	–	–	30	2.6
ECC	560	442	–	610	235	0.25	2.00 (PVA)	14.6	62	5.9
UHPC	900	1005	–	–	220	0.22	2.00 (Steel)	40.3	126	8.8

The column specimens were strengthened using a 15 mm-thick ECC or UHPC jacket. To ensure adequate placement of the thin strengthening layer and proper fiber dispersion, the fresh properties of the materials were evaluated. NC exhibited a slump of approximately 90 mm, while the ECC and UHPC mixtures demonstrated flow values of about 650 mm and 720 mm, respectively, indicating sufficient workability to achieve proper casting and effective bonding with the existing concrete surface.

**Fig. 2:**
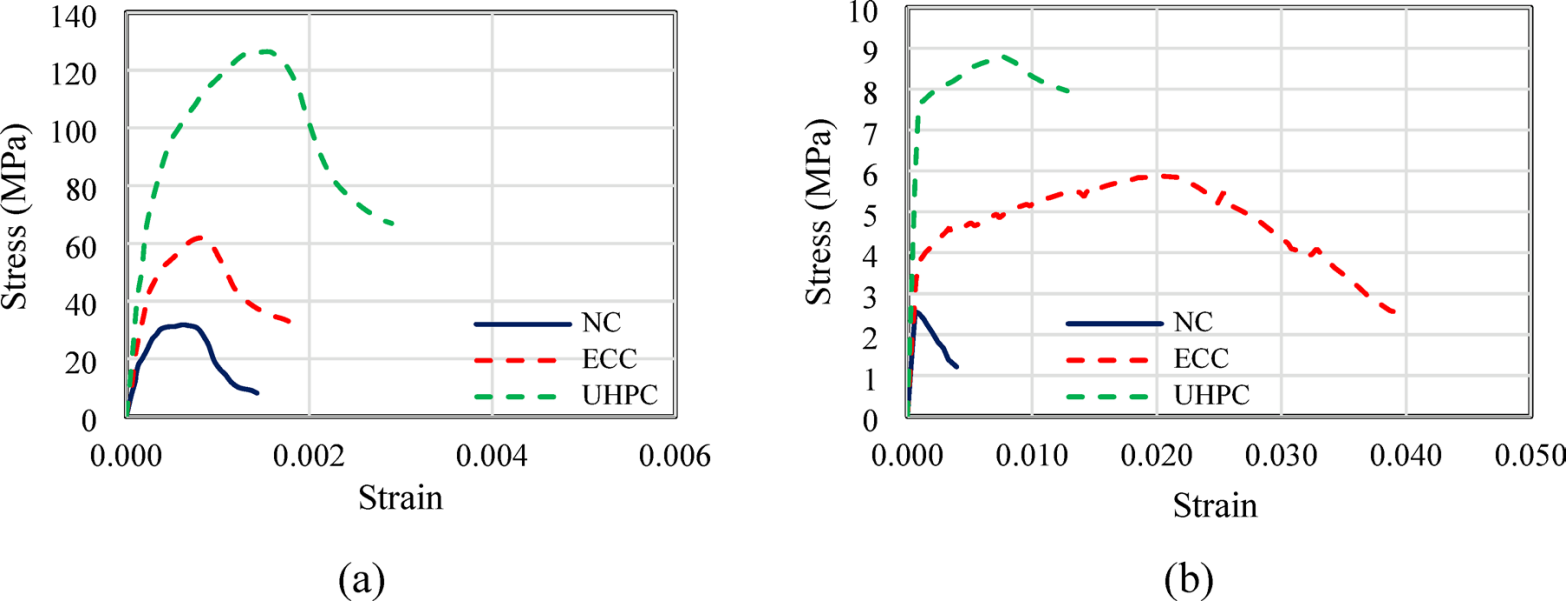
Stress–strain relationships for used concrete: (**a**) in compression and (**b**) in tension.

Standardized concrete cylinders with a diameter of 150 mm and a height of 300 mm were cast for each concrete mix and subsequently cured. Their compressive strength was then evaluated in accordance with ASTM C39/C39M ^38^. Three specimens per mix were tested, and the average compressive strength values are summarized in Table 2. To assess the tensile strength of the different concrete types, dog-bone specimens were subjected to uniaxial tensile testing following ASTM D3039/3039M ^39^. The measured tensile strength values were 2.6 MPa for NC, 5.9 MPa for ECC, and 8.8 MPa for UHPC, as shown in Fig. 2. According to the manufacturer’s specifications, the steel reinforcement had yield strengths of 319 MPa and 372 MPa, ultimate strengths of 512 MPa and 576 MPa, and moduli of elasticity of 205 GPa and 208 GPa for smooth and deformed steel bars, respectively.

### Preparation and casting of specimens

**Fig. 3:**
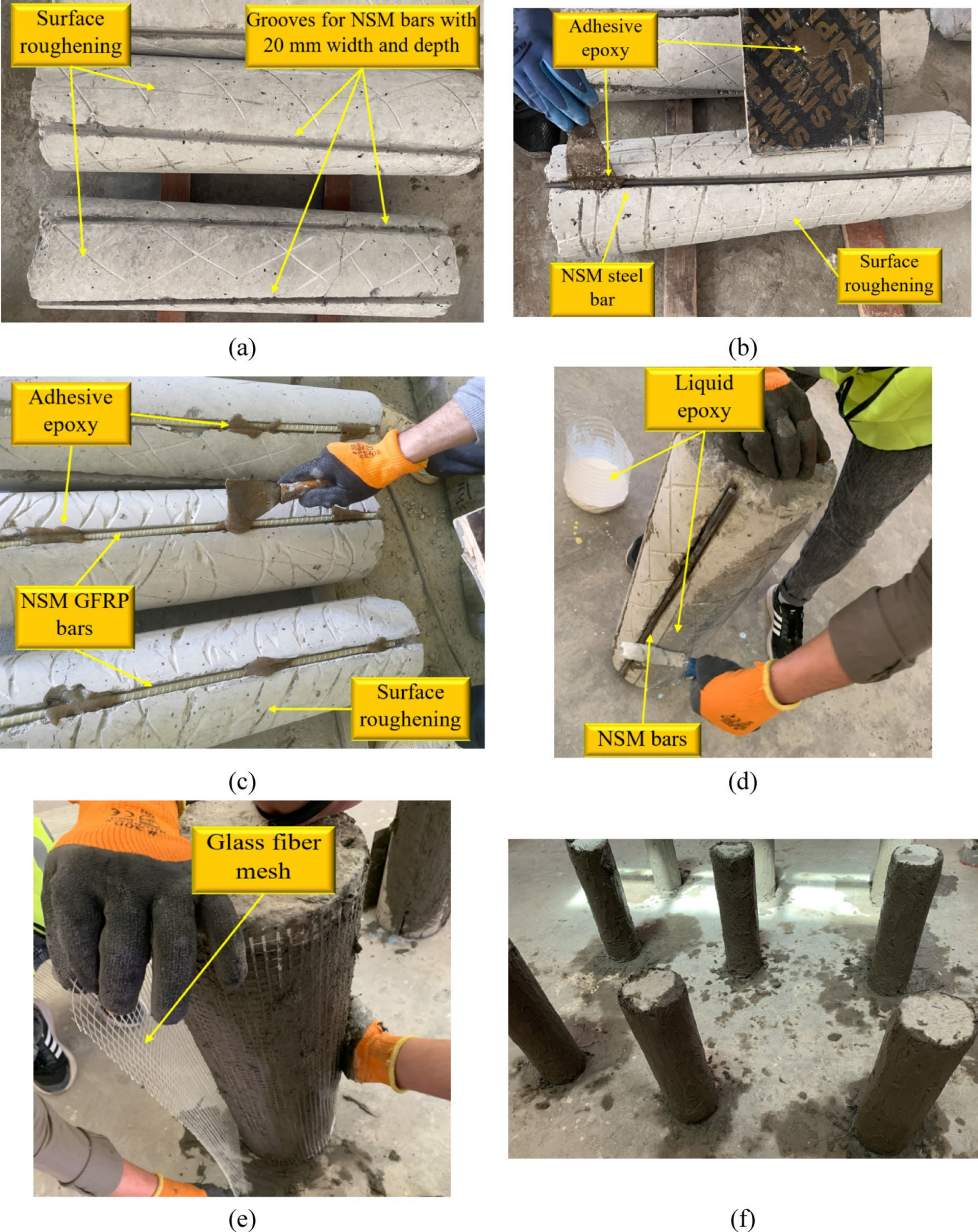
Strengthening procedures: (**a**) surface roughening and grooves cutting, (**b**) NSM steel bars application, (**c**) NSM GFRP bars application, (**d**) application of liquid epoxy to column surface, (**e**) installation of high-performance concrete and GFRP mesh, and (**f**) tested columns.

Figure 3 illustrates the step-by-step process of casting and preparing circular columns that were strengthened with NSM bars and confined with an outer layer incorporating GFRP mesh. Initially, as displayed in Fig. 3a, the column surface was adapted by roughening and cleaning it to ensure proper adhesion, followed by marking and positioning grooves to accommodate the NSM bars. In Fig. 3b, NSM steel bars were installed into the pre-cut grooves using epoxy adhesive, while Fig. 3c depicts the placement of NSM GFRP bars in a similar manner. Once the reinforcement was secured, liquid epoxy was applied to the column’s surface, as shown in Fig. 3d, to improve the bond between the existing concrete and the subsequent strengthening layers. Figure 3e demonstrates the application of a HPC overlay combined with a GFRP mesh, which was carefully wrapped around the column to provide additional confinement and improve structural performance. The GFRP mesh was wrapped around the column and embedded within the ECC/UHPC strengthening layer. The mesh was not pre-tensioned; instead, it was placed around the column with an overlap length of 200 mm to ensure continuity of the confinement layer. During casting of the ECC/UHPC jacket, the mesh was properly fixed to maintain its position and to ensure full embedding within the cementitious matrix. Finally, Fig. 3f presents all the tested columns after the strengthening process was completed.

### Instrumentation and testing of specimens

**Fig. 4:**
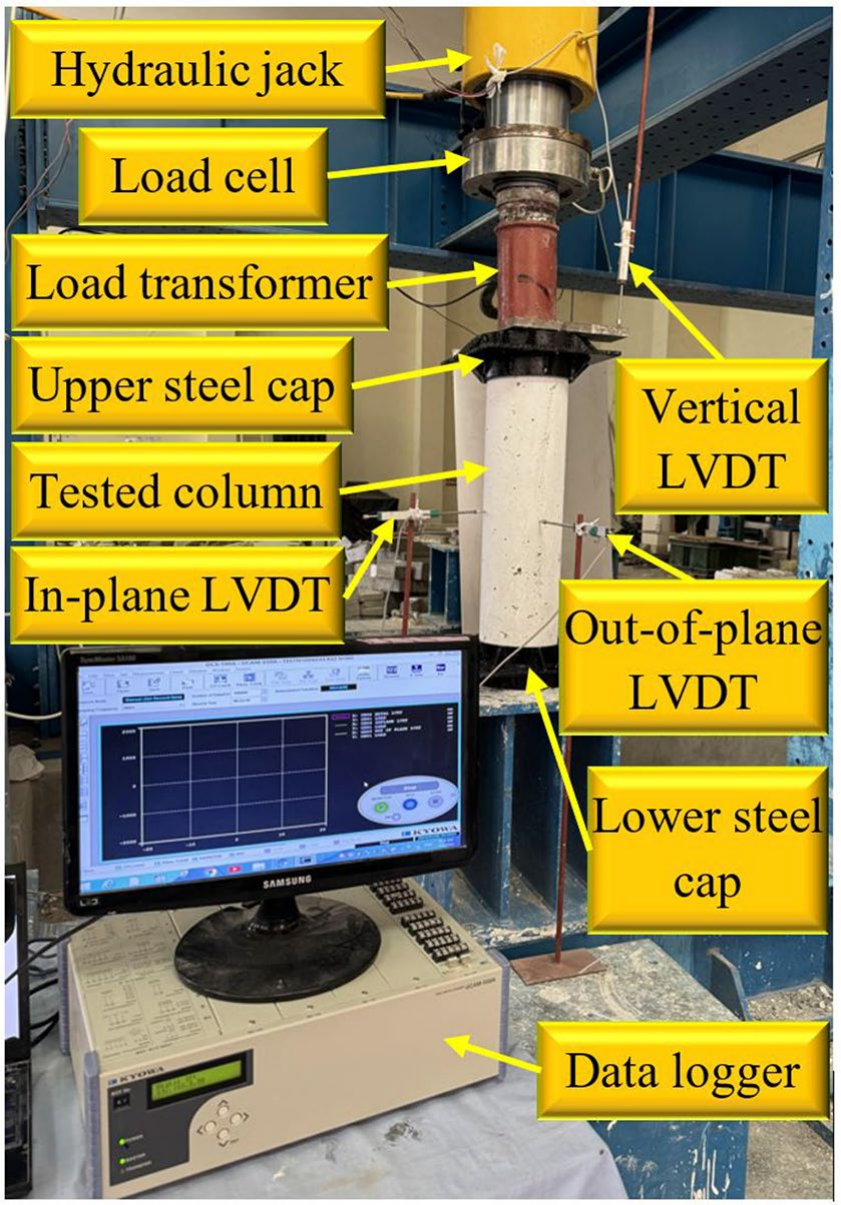
Test setup for all tested columns.

Figure 4 displays the experimental setup and instrumentation employed for the axial testing of the column specimens. The test was conducted using a hydraulic jack positioned at the top to apply a compressive load through a load cell, which accurately measured the applied force. A load transformer was incorporated to ensure uniform load distribution across the column. The column was secured between an upper and lower steel cap, facilitating proper load transfer and boundary conditions. To monitor displacement and stability during loading, three linear variable differential transformers (LVDTs) were strategically placed: a vertical LVDT to measure axial displacement, an in-plane LVDT to capture lateral displacements along one axis, and an out-of-plane LVDT to assess displacements perpendicular to the in-plane measurement. A data logger was connected to the system to continuously record load and displacement data throughout the tests. The combination of these components ensured precise monitoring of the specimens’ structural response under axial loading.

## Experimental results

### Failure patterns

**Fig. 5:**
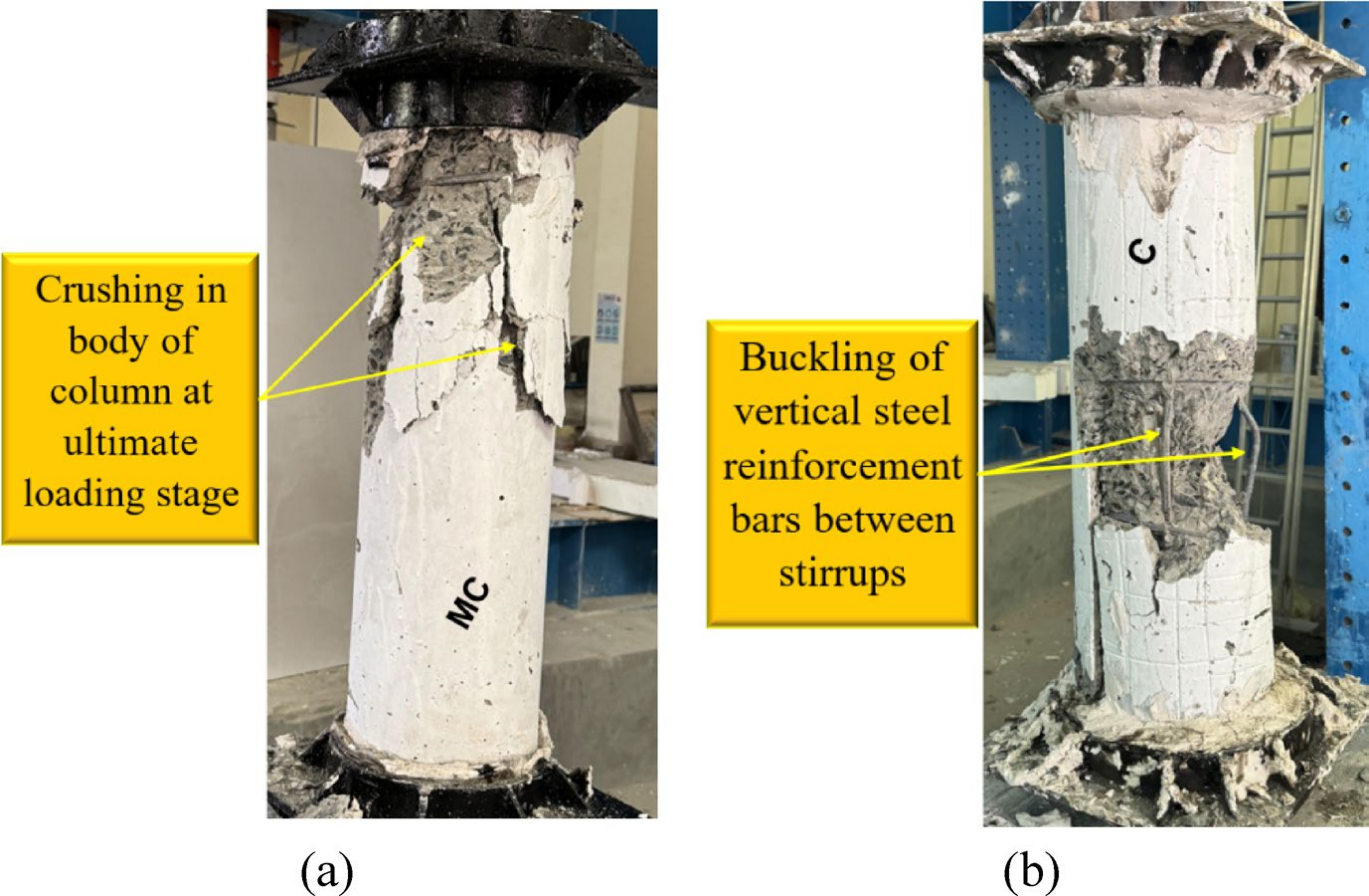
Failure of G1: (**a**) column MC and (**b**) column C^40^.

**Fig. 6:**
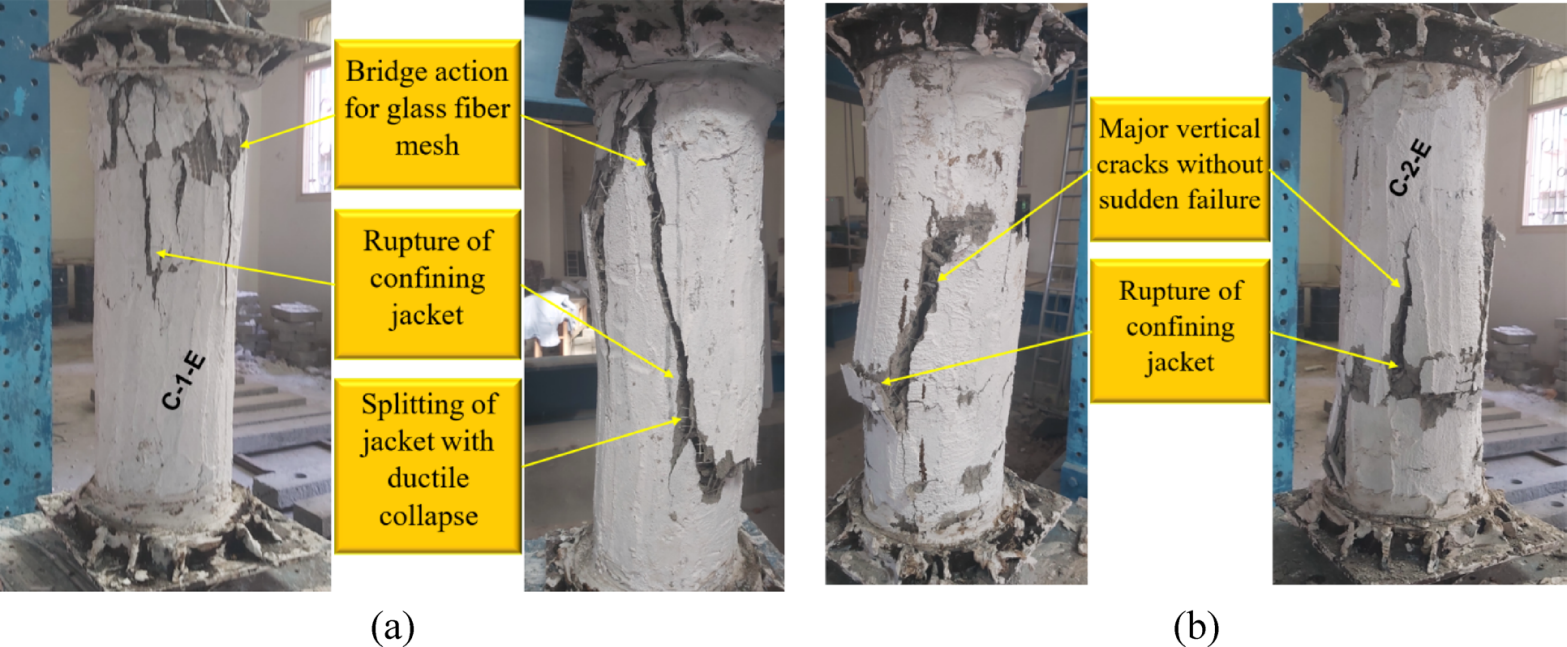
Failure of G2: (**a**) column C-1-E and (**b**) column C-2-E.

**Fig. 7:**
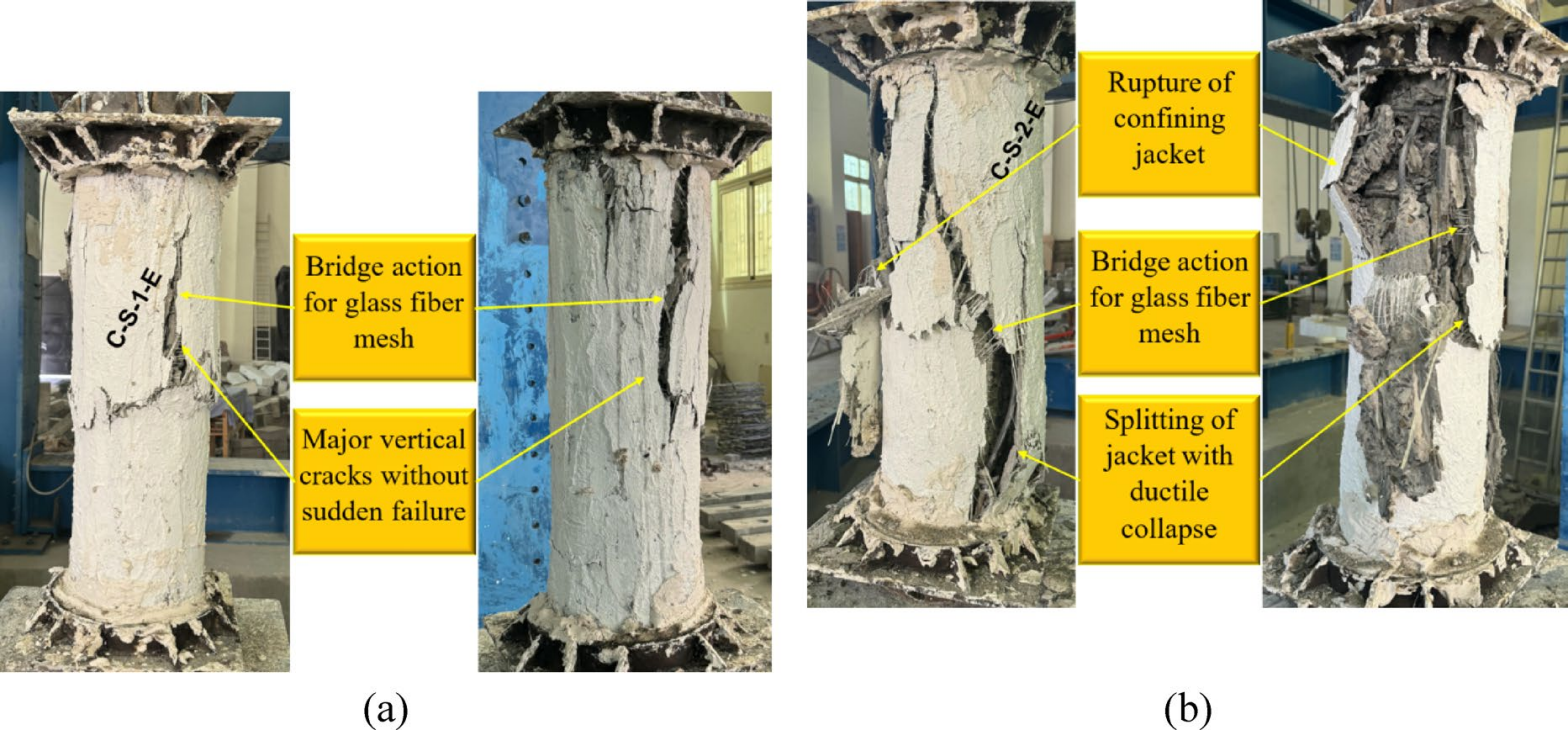
Failure of G3: (**a**) column C-S-1-E and (**b**) column C-S-2-E.

**Fig. 8:**
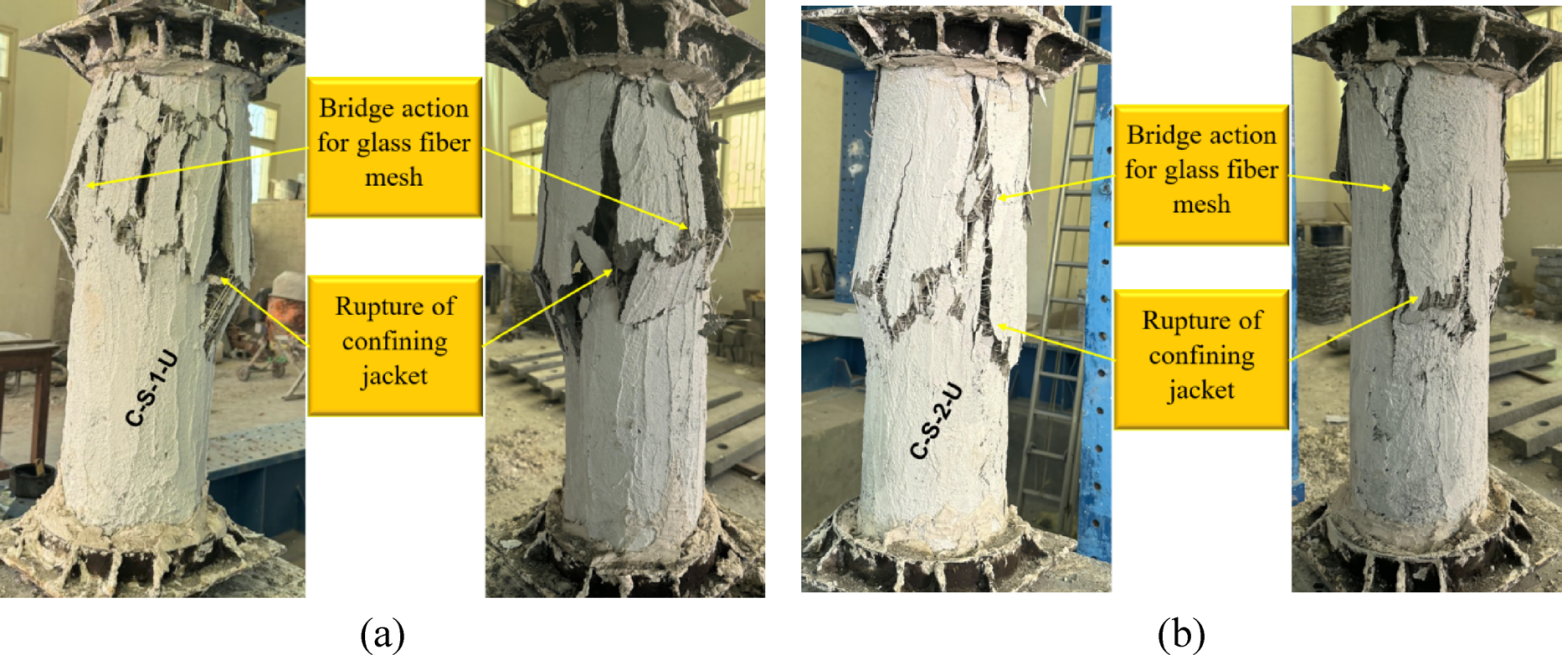
Failure of G4: (**a**) column C-S-1-U and (**b**) column C-S-2-U.

**Fig. 9:**
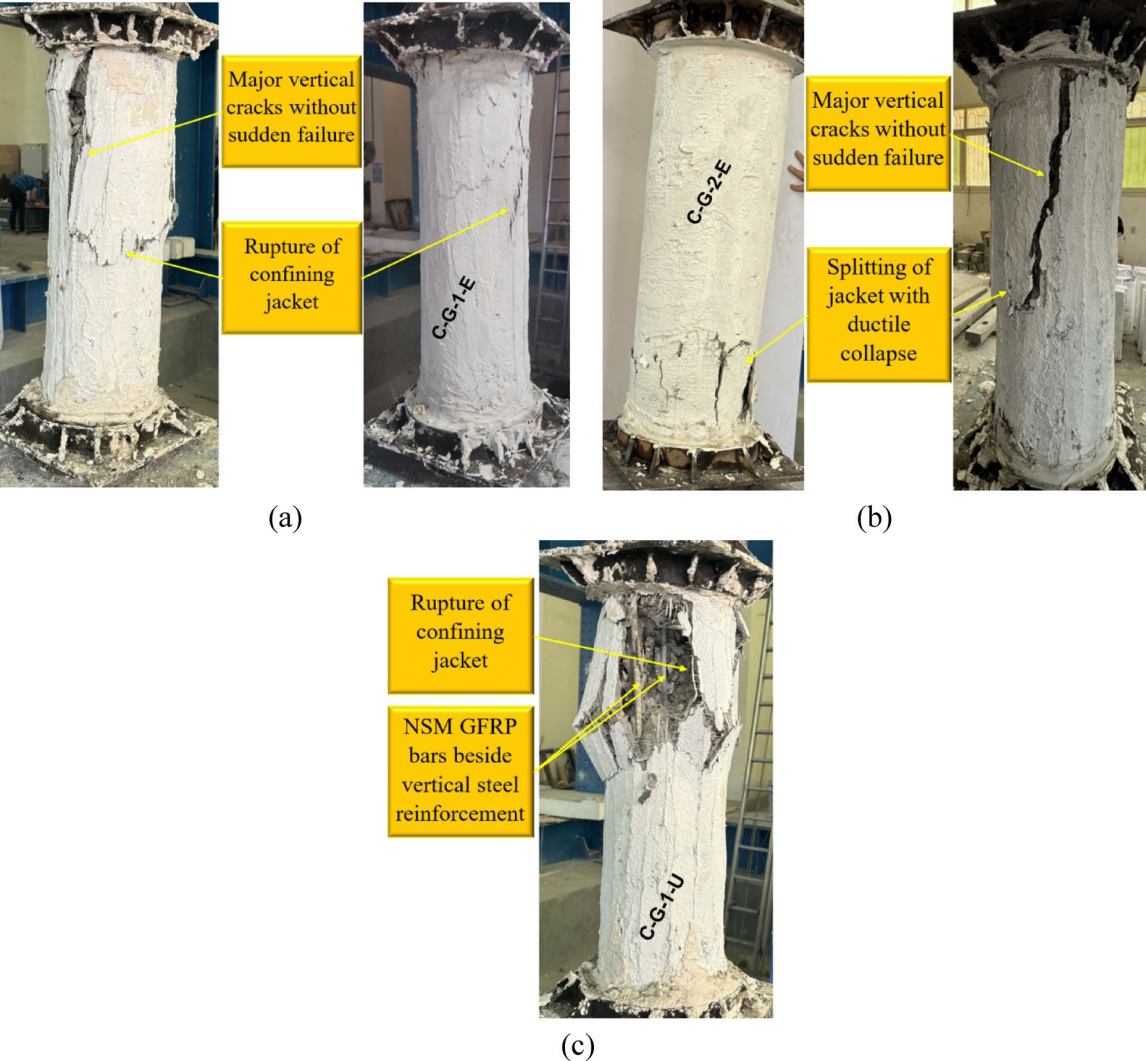
Failure mode of G5: (**a**) column C-G-1-E, (**b**) column C-G-2-E, and (**c**) column C-G-1-U.

Figures 5–9 illustrate the failure modes of the tested columns subjected to axial compression. Figure 5 presents the failure characteristics of the control group, where column MC, serving as the master column, exhibited concrete crushing at the ultimate loading stage. In contrast, column C, which had a reduced reinforcement ratio to simulate the effects of steel corrosion, experienced a more severe failure mode characterized by the buckling of the main reinforcement bar between stirrups. This indicates that the reinforcement reduction compromised the column’s load capacity and led to premature failure. The tested MC and C columns in this group were adopted from the experimental study conducted by Shahin et al. ^40^.

Figure 6 depicts the failure modes of the strengthened columns in the second group, which were externally confined with an ECC jacket incorporating GFRP mesh. Specimen C-1-E was strengthened with a single layer of GFRP mesh, while specimen C-2-E incorporated two layers. The failure patterns of these columns demonstrated improved ductility compared with the unstrengthened counterparts. The strengthening system effectively delayed the onset of brittle failure by providing confinement and mitigating crack propagation. In specimen C-1-E, failure was characterized by jacket splitting and rupture of the confining layer, while the presence of the GFRP mesh contributed to a bridging action that enhanced structural integrity. Specimen C-2-E, which had two layers of GFRP mesh, exhibited major vertical cracks but without sudden failure, suggesting that the additional confinement further improved the column’s ability to withstand higher loads before rupture. These findings highlight the effectiveness of the ECC-GFRP strengthening system in enhancing both the strength and ductility of deficient columns under axial loading.

Figures 7 and 8 illustrate the failure modes of the third and fourth groups, which were strengthened using a combination of NSM steel bars and an external confinement jacket. The third group (Fig. 7) utilized an ECC jacket, while the fourth group (Fig. 8) incorporated a UHPC jacket. The failure modes of the ECC-confined specimens in the third group demonstrated significant improvements in ductility and load resistance compared with unconfined columns. In specimen C-S-1-E, where a single layer of GFRP mesh was used, failure was characterized by major vertical cracks without sudden collapse, indicating that the external jacket effectively redistributed stresses. The presence of GFRP contributed to a bridging action, which mitigated crack propagation. On the other hand, specimen C-S-2-E, which had two layers of GFRP mesh, exhibited further improvements in ductility, with visible splitting of the jacket occurring in a more controlled manner. The additional GFRP layers enhanced the confinement effectiveness, delaying sudden failure and enabling a more gradual reduction in load capacity before collapse.

Similarly, in the fourth group (Fig. 8), where a UHPC jacket was employed, the specimens exhibited improved structural performance compared with unstrengthened columns. Specimen C-S-1-U, with one GFRP layer, displayed rupture of the confining jacket and the activation of GFRP bridging action, similar to its ECC counterpart. However, the use of UHPC resulted in a more rigid confinement, leading to localized failure rather than distributed cracking. Specimen C-S-2-U, with two layers of GFRP, demonstrated enhanced confinement, with more extensive jacket rupture but without abrupt failure. The superior mechanical properties of UHPC contributed to higher crack resistance, but the overall failure mode remained consistent with those observed in the ECC-confined specimens.

Overall, the application of NSM steel bars in combination with external confinement notably improved the structural integrity of the columns. The use of ECC and UHPC as jacketing materials enhanced the capacity and ductility, while the incorporation of GFRP mesh further strengthened the system by mitigating sudden failure. The observed failure patterns confirm that a multi-layered strengthening approach effectively enhances the structural resilience of deficient columns under axial loading.

The failure modes of the last group (G5) are illustrated in Fig. 9, where the specimens were strengthened using NSM GFRP bars instead of steel, as in the previous groups. The failure of specimen C-G-1-E exhibited major vertical cracks accompanied by rupture of the confining jacket, similar to the behavior observed in ECC-confined columns from previous groups. The presence of NSM GFRP bars contributed to the redistribution of stresses and enhanced the column’s ductility, delaying the onset of sudden failure. The single-layer GFRP mesh in the ECC jacket provided a moderate level of confinement, allowing for a controlled failure mode characterized by progressive cracking rather than abrupt collapse.

In specimen C-G-2-E, where two layers of GFRP mesh were incorporated within the ECC jacket, failure occurred through splitting in the jacket with ductile collapse. Compared with C-G-1-E, the additional GFRP reinforcement enhanced the confinement effect, resulting in improved structural integrity and more gradual failure propagation. The major vertical cracks observed suggest that the external jacket effectively contained internal damage, preventing catastrophic failure.

For specimen C-G-1-U, which was strengthened with a UHPC jacket containing a single layer of GFRP mesh, failure was characterized by the rupture of the confining jacket along with localized damage near the NSM GFRP bars. The UHPC provided a stiffer confinement compared with ECC, leading to more brittle jacket failure while still maintaining sufficient ductility to avoid sudden collapse. The use of NSM GFRP bars alongside vertical steel reinforcement appeared to influence the failure mode by providing additional tensile resistance, thus improving the load capacity. During the experimental tests, no noticeable slip or debonding was observed between the ECC/UHPC strengthening layer and the original concrete surface. This indicates that the adopted surface preparation procedure provided adequate bonding, allowing the strengthening layer to act compositely with the concrete core during loading.

The replacement of NSM steel bars with NSM GFRP bars resulted in distinct failure patterns compared with the previous groups. The ECC-confined specimens showed a more ductile failure pattern, while the UHPC-confined specimens exhibited higher rigidity with localized rupture. The presence of multiple layers of GFRP mesh further contributed to the structural resilience, with two-layer configurations providing better confinement than single-layer ones. These findings highlight the effectiveness of combining NSM GFRP bars with external confinement in enhancing the performance of deficient columns under axial loading.

### Load-vertical displacement

**Fig. 10:**
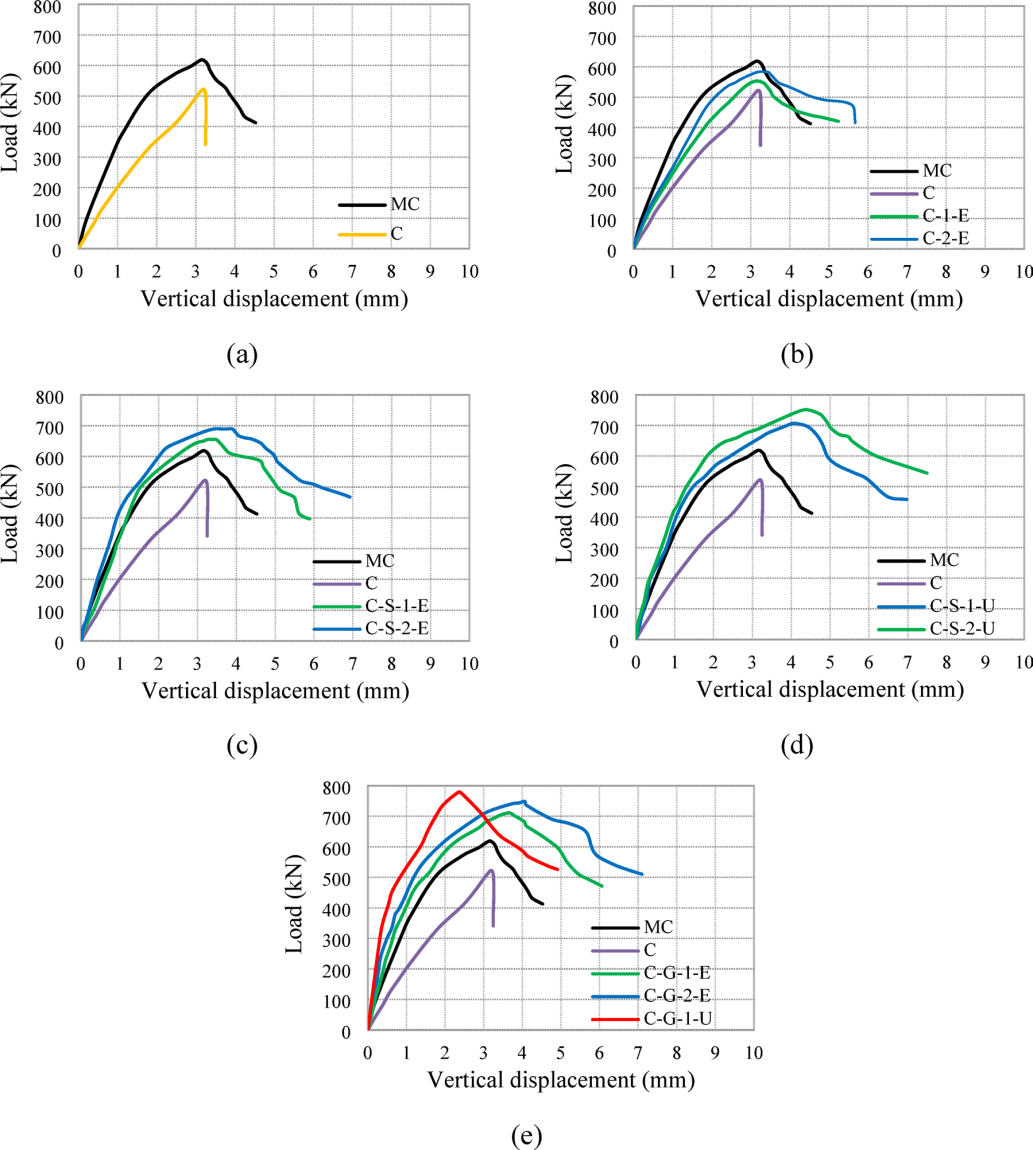
Load-vertical displacement relationships for all tested columns: (**a**) G1, (**b**) G2, (**c**) G3, (**d**) G4, and (**e**) G5.

The load versus vertical displacement behavior of all tested columns is illustrated in Fig. 10, while the ultimate load capacities and absorbed energy are summarized in Table 3. The control group, exhibited a significant reduction in axial load capacity for specimen C owing to the reduced reinforcement bars ratio, simulating the effect of steel corrosion. The failure of specimen C was brittle, with a severe decline in load capacity after achieving its peak.

**Table 3:** Test outcomes.

Column	Ultimate stage				Absorbed energy (E) (kN.mm)	E/E_C_	E/E_MC_
	P_u_ (kN)	P_u_/P_uC_	P_u_/P_uMC_	Δ_Pu_ (mm)			
MC	618	1.18	1.00	3.14	1999	2.14	1.00
C	522	1.00	0.84	3.19	933	1.00	0.47
C	522	1.00	0.84	3.19	933	1.00	0.47
C-1-E	553	1.06	0.89	3.16	2032	2.18	1.02
C-2-E	583	1.12	0.94	3.24	2465	2.64	1.23
C	522	1.00	0.84	3.19	933	1.00	0.47
C-S-1-E	655	1.25	1.06	3.48	2857	3.06	1.43
C-S-2-E	689	1.32	1.11	3.41	3704	3.97	1.85
C	522	1.00	0.84	3.19	933	1.00	0.47
C-S-1-U	705	1.35	1.14	4.15	3677	3.94	1.84
C-S-2-U	751	1.44	1.22	4.36	4388	4.70	2.20
C	522	1.00	0.84	3.19	933	1.00	0.47
C-G-1-E	711	1.36	1.15	3.64	3242	3.47	1.62
C-G-2-E	748	1.43	1.21	4.06	4162	4.46	2.08
C-G-1-U	779	1.49	1.26	2.35	2877	3.08	1.44

P_u_: Ultimate load; P_uC_: Ultimate load for deficient column; P_uC_: Ultimate load for master column; Δ_Pu_: Vertical displacement recorded at P_u_; E: Absorbed energy.

**Fig. 11:**
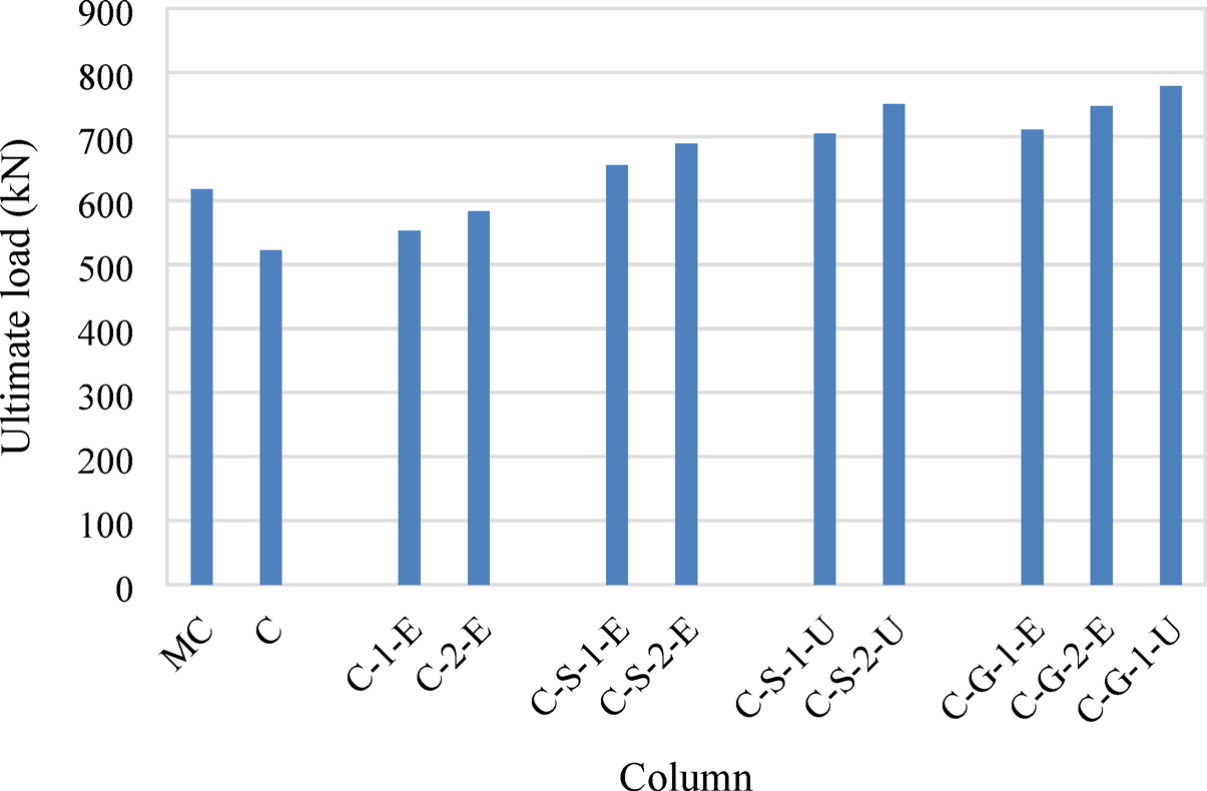
Load capacities of tested columns.

The second group, in which specimens were strengthened using an external ECC jacket with embedded GFRP mesh, exhibited improved load capacities compared with the control group, as displayed in Fig. 11. The ultimate load increased by 6% (553 kN) and 12% (583 kN) for specimens C-1-E and C-2-E, respectively. The presence of GFRP mesh enhanced confinement, reducing the brittleness of failure and leading to a more gradual post-peak load deterioration.

**Fig. 12:**
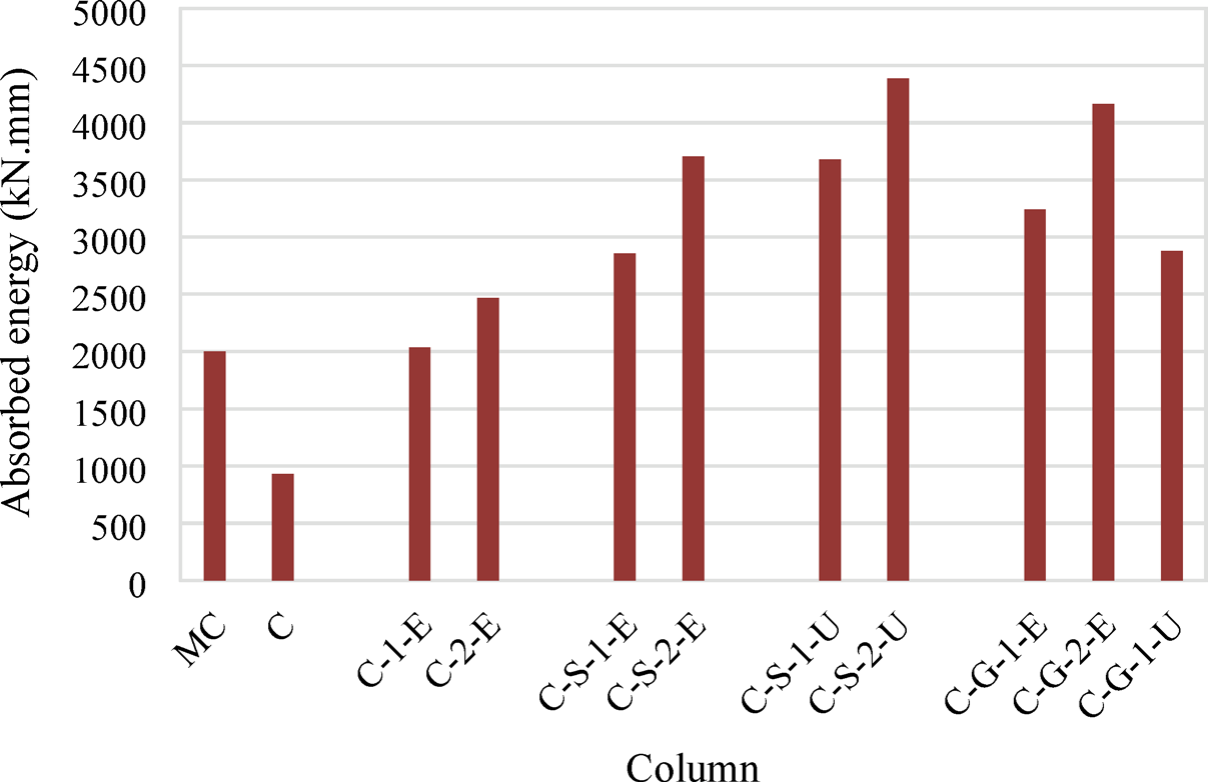
Absorbed energies of tested columns.

For the third group, which incorporated NSM steel bars and external ECC confinement, significant improvements in both load capacity and displacement characteristics were observed. The ultimate loads of specimens C-S-1-E and C-S-2-E were 25% (655 kN) and 32% (689 kN) higher than that of specimen C, respectively. The additional confinement provided by ECC and the inclusion of GFRP mesh improved both strength and ductility, allowing the columns to sustain higher loads with reduced brittle failure tendencies. The absorbed energy for these specimens was 2857 kN.mm and 3704 kN.mm, respectively, which were considerably higher than that of the control specimen (933 kN.mm), indicating enhanced energy dissipation, as illustrated in Fig. 12.

The fourth group utilized NSM steel bars but replaced the ECC jacket with UHPC. This modification led to further enhancements in axial load capacity and energy absorption. Specimens C-S-1-U and C-S-2-U demonstrated ultimate load increases of 35% (705 kN) and 44% (751 kN), respectively, compared with specimen C. The UHPC jacket provided superior confinement, delaying the onset of failure and allowing for greater energy dissipation, with absorbed energy values of 3677 kN.mm and 4388 kN.mm, respectively. The increased energy absorption (up to 4.70 times that of the control column) signifies improved ductility and post-peak behavior.

Finally, the fifth group incorporated NSM GFRP bars instead of steel bars along with ECC and UHPC jacketing. Specimens C-G-1-E and C-G-2-E, which used ECC jackets with one and two GFRP mesh layers, showed ultimate load increases of 36% (711 kN) and 43% (748 kN), respectively. Specimen C-G-1-U, with a UHPC jacket and a single GFRP layer, exhibited the highest improvement, with an ultimate load 49% (779 kN) greater than that of specimen C. The use of NSM GFRP bars, in conjunction with high-performance jacketing, enhanced both strength and energy absorption, with an absorbed energy value reaching 2877 kN.mm for C-G-1-U, indicating superior performance in terms of structural resilience (Fig. 12).

Overall, the load-displacement curves show that strengthening using NSM bars and external jacketing effectively improves column performance. ECC jacketing with GFRP mesh enhances both strength and ductility, while UHPC provides superior confinement, leading to the highest strength gains. The inclusion of NSM GFRP bars further contributes to enhanced structural performance, making them a viable alternative to traditional steel reinforcement in retrofitting applications.

## Finite element investigation

### Geometry and interaction characteristics of models

**Fig. 13:**
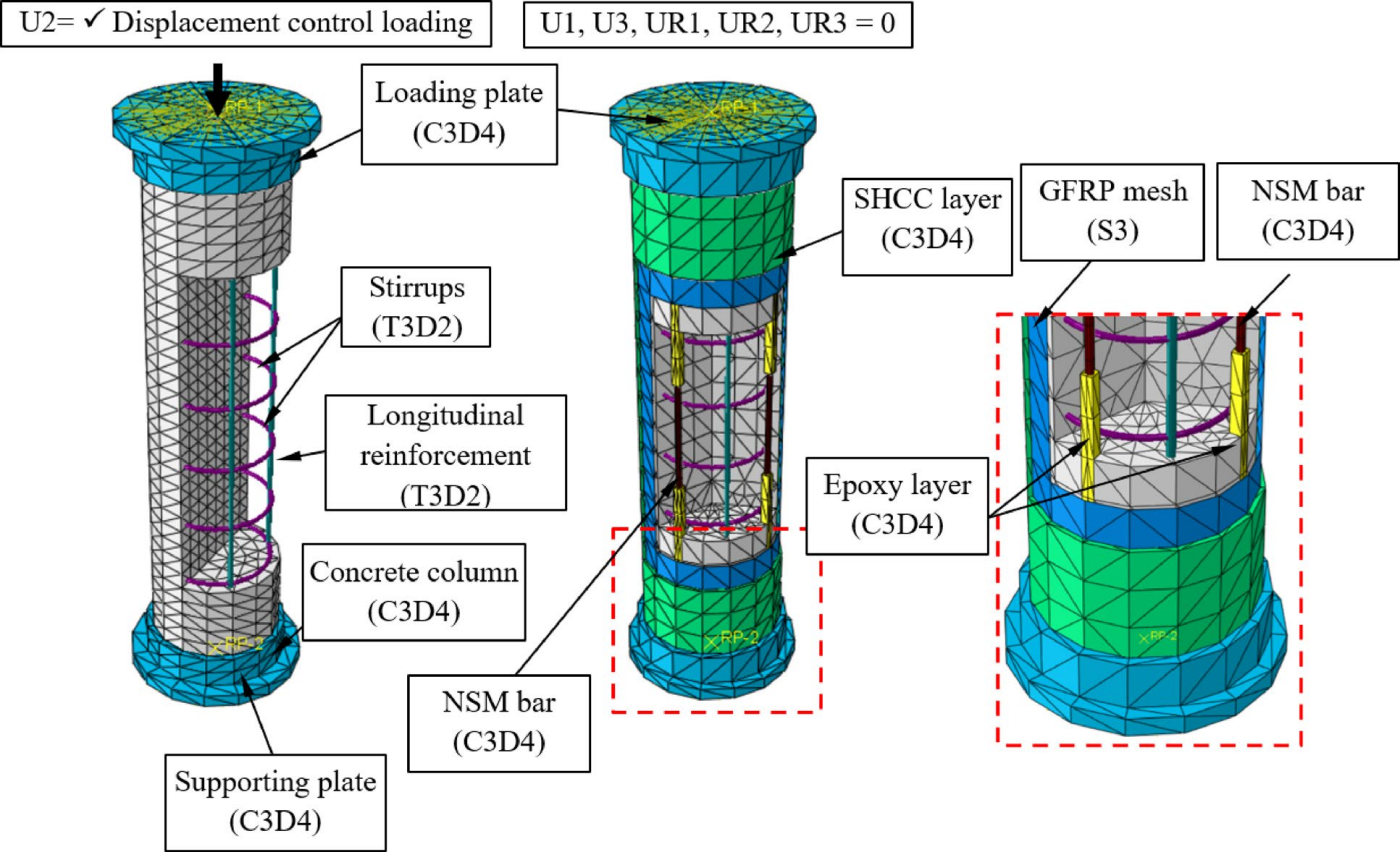
Numerical models of columns MC and C-S-1-E.

**Fig. 14:**
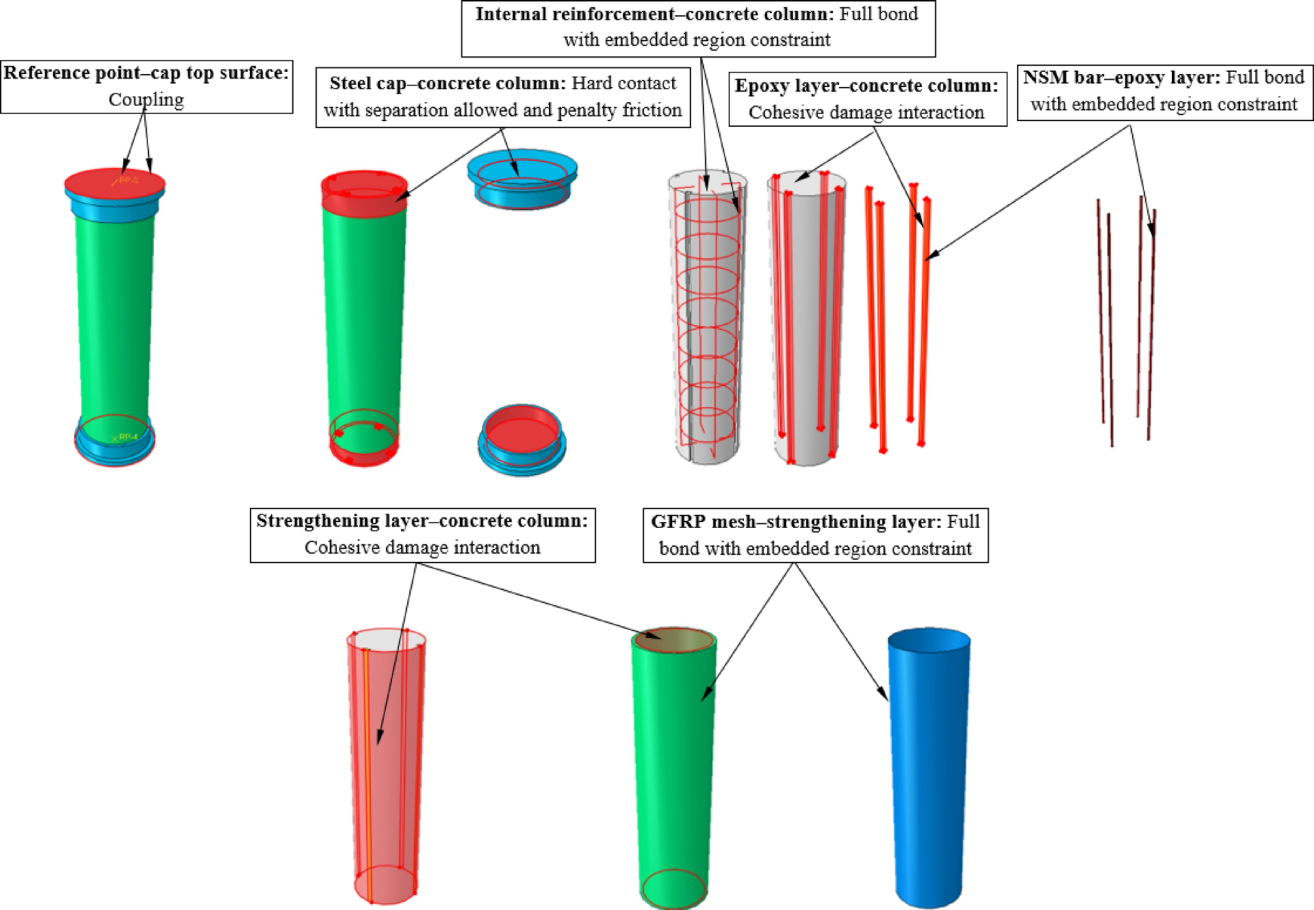
Interaction between different elements.

The numerical models of the tested columns were developed to simulate their structural response under axial compression (Figs. 13 and 14). The models were constructed using the finite element software Abaqus, incorporating nonlinear material behavior and detailed interaction properties to accurately represent the experimental conditions.

The geometric configuration of the columns followed the experimental setup. The main column body was modeled using C3D4 (four-node tetrahedral elements), ensuring an efficient balance between accuracy and computational cost. Reinforcement bars, including longitudinal steel bars and stirrups, were simulated through T3D2 (three-node truss elements) with embedded region constraints. Strengthening layers, including ECC and UHPC jackets, were modeled as separate C3D4 elements, while NSM reinforcement bars were explicitly defined.

The finite element model incorporated various interaction properties to simulate realistic bonding and slip conditions between different materials (Fig. 14). A full bond assumption was applied between the internal reinforcement and the concrete column using an embedded region constraint, ensuring perfect adhesion between the steel reinforcement and the concrete matrix. Similarly, NSM bars and the epoxy layer were modeled with full bond using embedded region constraints, assuming sufficient anchorage without significant slip. To take possible debonding at the interface into consideration, a cohesive damage interaction was assigned between the epoxy layer and the concrete column. The interaction between the steel cap and the concrete column was defined utilizing hard contact with separation allowed and penalty friction to accurately represent the load transfer mechanism. For strengthening layers, cohesive damage interaction was employed between the ECC or UHPC jackets and the concrete column to capture potential debonding or delamination effects. Finally, a full bond was assumed between the GFRP mesh and the strengthening layer through an embedded region constraint, ensuring proper load transfer.

The columns were constrained to simulate experimental conditions accurately. The bottom support plate was fully fixed to restrict all degrees of freedom, while the top loading plate was assigned a reference point coupled to its surface to apply axial displacement in a controlled manner. Contact interactions between the loading plate and the column were defined to allow separation and frictional slip, simulating real test conditions. The numerical models successfully replicated the experimental failure modes and load-displacement responses observed in the tests. The adopted material models and interaction properties provided a robust framework for analyzing the strengthening techniques used in this study.

### Materials constitutive modeling

The elastic behavior of NC, ECC, and UHPC was modeled using a Poisson’s ratio of 0.2. The modulus of elasticity was established according to ACI 318 ^41^. The concrete damage plasticity (CDP) approach was employed to characterize the nonlinear response of concrete due to compressive and tensile stresses, as it effectively captures deterioration caused by cracking and nonlinear deformations ^42^. This model incorporates isotropic damage elasticity and nonlinear behavior in both compression and tension, providing an accurate representation of concrete’s plastic response under various loading conditions.

The Carreira-Chu model ^43^, described by Eqs. 1 and 2, was adopted to capture the compressive stress–strain behavior of NC. To represent the distinct behavior of ECC and UHPC, previously established stress–strain relationships from Zhou et al. ^44^, presented in Eqs. 3 and 4, were utilized. A bilinear elastic–plastic model with hardening was employed to simulate the behavior of reinforcement bars and the anchorage system.

1 $${\upsigma}_{\mathrm{c}}={\mathrm{f}}_{\mathrm{c}}\left[\frac{\propto \left(\frac{{\upvarepsilon}_{\mathrm{c}}}{{\upvarepsilon}_{\mathrm{c}0}}\right)}{\propto -1+{\left(\frac{{\upvarepsilon}_{\mathrm{c}}}{{\upvarepsilon}_{\mathrm{c}0}}\right)}^{\propto }}\right]$$

2 $${\upsigma}_{\mathrm{t}}=\left\{\begin{array}{lll}{\mathrm{f}}_{\mathrm{t}}\left[1.2\frac{{\upvarepsilon}_{\mathrm{t}}}{{\upvarepsilon}_{\mathrm{t}0}}-0.2{\left(\frac{{\upvarepsilon}_{\mathrm{t}}}{{\upvarepsilon}_{\mathrm{t}0}}\right)}^{6}\right] \quad 0\le {\upvarepsilon}_{\mathrm{t}}\le {\upvarepsilon}_{\mathrm{t}0}\\ \\ {\mathrm{f}}_{\mathrm{t}} \left[\frac{\frac{{\upvarepsilon}_{\mathrm{c}}}{{\upvarepsilon}_{0}}}{1.25{\left(\frac{{\upvarepsilon}_{\mathrm{t}}}{{\upvarepsilon}_{\mathrm{t}0}}-1\right)}^{2}-\frac{{\upvarepsilon}_{\mathrm{t}}}{{\upvarepsilon}_{\mathrm{t}0}}}\right] \quad \quad \quad {\upvarepsilon}_{\mathrm{t}0}<{\upvarepsilon}_{\mathrm{t}}\end{array}\right.$$

This equation integrates essential material properties of concrete to predict its stress–strain behavior, which is vital for assessing the performance of RC columns. It establishes a relationship between the concrete stress (σ_c_) and its corresponding strain (ε_c_), considering two critical aspects: the peak strength and the post-peak response. The parameter f_c_ denotes the concrete’s peak stress, while ε_co_ indicates the strain at which this peak is reached. Additionally, the parameter α, assumed to be 2.5 in this study, characterizes the shape of the stress–strain response beyond the peak strength.

3 $${\mathrm{f}}_{\mathrm{c}}=\left\{\begin{array}{lll} \quad \quad {\mathrm{E}}_{0 }{\upvarepsilon}_{\mathrm{c}} \qquad  \qquad \qquad \qquad \qquad \qquad {\upvarepsilon}_{\mathrm{c}}\le {0.4 \upvarepsilon }_{\mathrm{c}\mathrm{p}}\\ \\ \\ {\mathrm{E}}_{0 }{\upvarepsilon}_{\mathrm{c}} \left(1-0.308\frac{{\mathrm{E}}_{0 }{\upvarepsilon}_{\mathrm{c}}}{{\mathrm{f}}_{\mathrm{c}}^{^{\prime}}}+0.124\right) \quad \quad {0.4 \upvarepsilon }_{\mathrm{c}\mathrm{p}}<{\upvarepsilon}_{\mathrm{c}}\le {\upvarepsilon}_{\mathrm{c}\mathrm{p}}\end{array}\right.$$

4 $${\mathrm{f}}_{\mathrm{t}}=\left\{\begin{array}{lll}\frac{{\mathrm{f}}_{\mathrm{t}\mathrm{c} }}{{\upvarepsilon}_{\mathrm{t}\mathrm{c}}}{\upvarepsilon}_{\mathrm{t}} \qquad \qquad \qquad \qquad  \qquad 0\le {\upvarepsilon}_{\mathrm{t}}\le { \upvarepsilon }_{\mathrm{t}\mathrm{c}}\\ \\ \\ {\mathrm{f}}_{\mathrm{t}\mathrm{c} }+ \frac{{\mathrm{f}}_{\mathrm{t}\mathrm{u}}-{\mathrm{f}}_{\mathrm{t}\mathrm{c}}}{{\upvarepsilon}_{\mathrm{t}\mathrm{u}}-{\upvarepsilon}_{\mathrm{t}\mathrm{c}}}\left(\upvarepsilon -{\upvarepsilon}_{\mathrm{t}\mathrm{c}}\right) \qquad   { \upvarepsilon }_{\mathrm{t}\mathrm{c}}\le {\upvarepsilon}_{\mathrm{t}}\le { \upvarepsilon }_{\mathrm{t}\mathrm{u}}\end{array}\right.$$

Several symbols are used in both Eqs. 4 and 5 to represent important material properties of the concrete under consideration. E₀ refers to the stiffness of the concrete, which quantifies its resistance to elastic deformation. ε_cp_ is the strain at maximum stress, marking the point at which the concrete reaches its maximum stress just prior to failure. f_tc_ represents the tensile strength of the concrete, indicating the highest stress the material can withstand under tension. ε_tc_ denotes the strain at first crack, representing the point at which the concrete begins to crack under stress. Additionally, f_tu_ stands for the ultimate tensile strength of the concrete, while ε_tu_ is the corresponding strain.

The material models presented in the previous subsection describe the constitutive behavior of each material individually. The confinement effect provided by the ECC/UHPC jacket and the corresponding confined concrete behavior were incorporated in the analysis using confinement equations adopted from the study conducted by Shahin et al. ^40^. These equations were used to account for the lateral confinement pressure acting on the concrete core due to the strengthening jacket.

To ensure consistency while remaining within predetermined parameter ranges, a sensitivity study was performed to calibrate the CDP model parameters for the various types of concrete. Important factors influencing the concrete response were examined, including material-specific dilation angles (30° for ECC and UHPC, and 25° for NC). A second stress invariant proportion of 0.66 and a yield stress proportion of 1.16 were identified as optimal values. Furthermore, the assumption of a non-viscous material (μ = 0.0) and the defaulting eccentricity (e = 0.1) were found to be appropriate for realistic simulations. These modifications, combined with the optimal mesh size of 10 mm identified (as detailed in studies ^45–47^ on mesh size selection), enable faster and more accurate simulations that capture a wide range of concrete behaviors under different stress conditions.

### Model verification

**Fig. 15:**
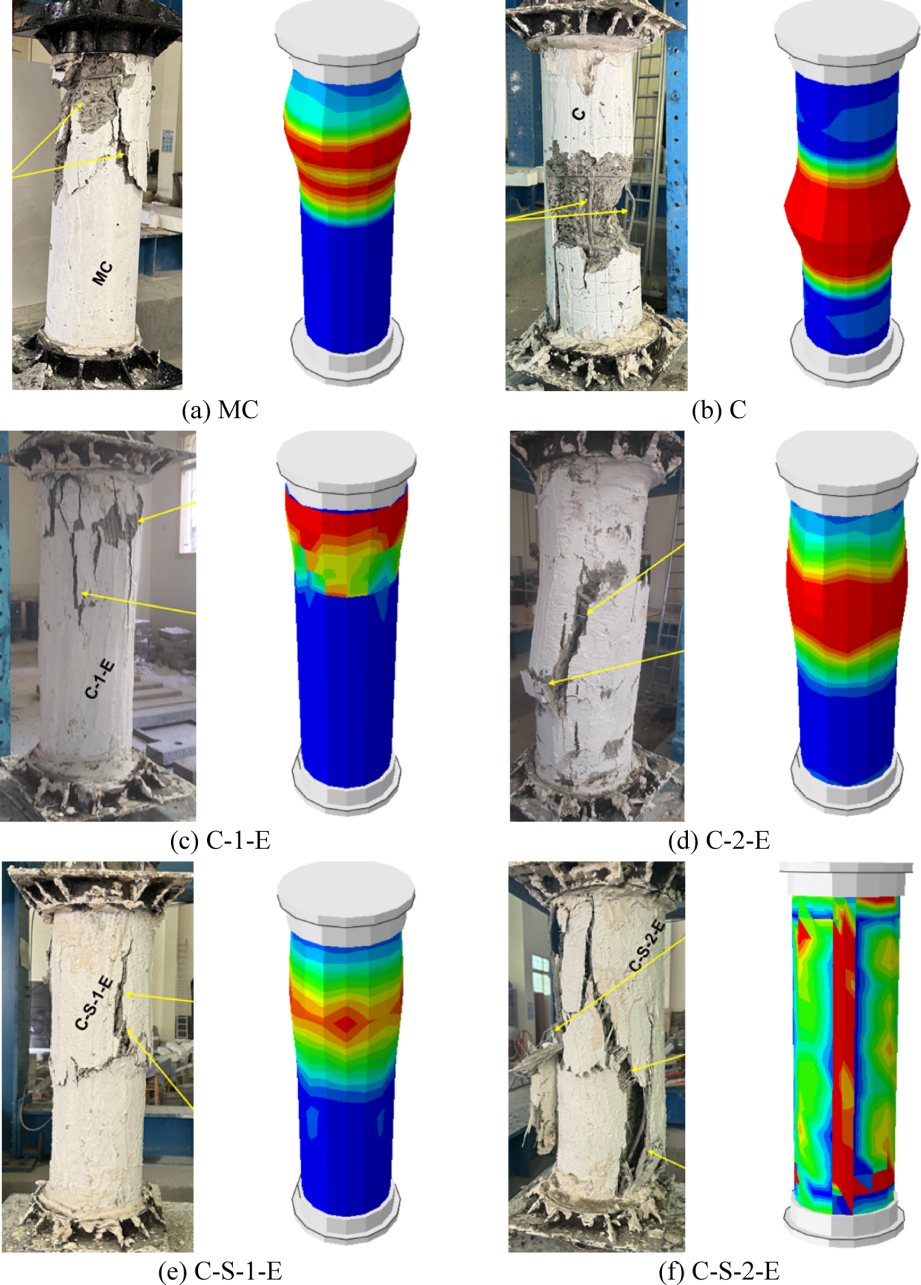

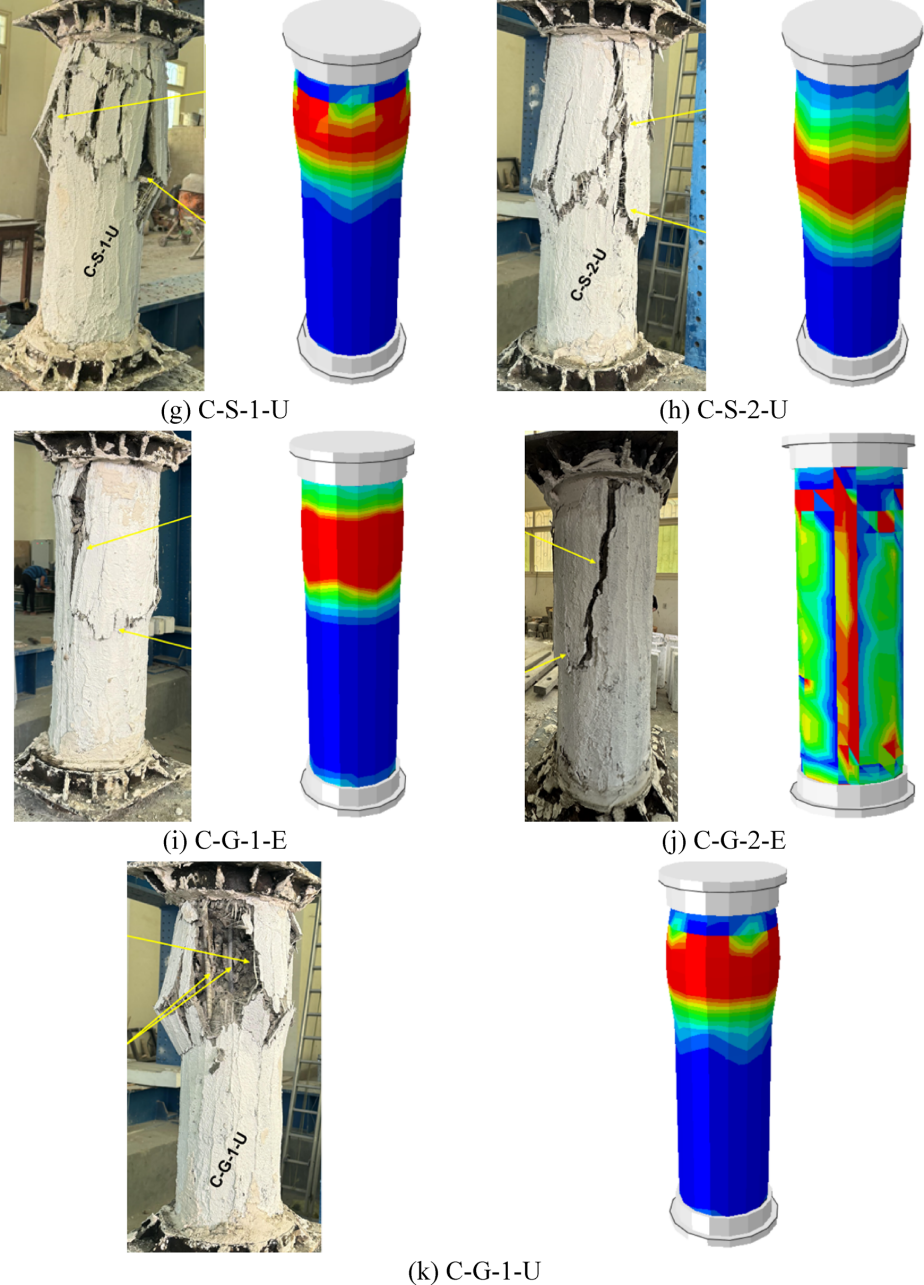
Comparison of experimental and numerical collapse patterns.

**Fig. 16:**
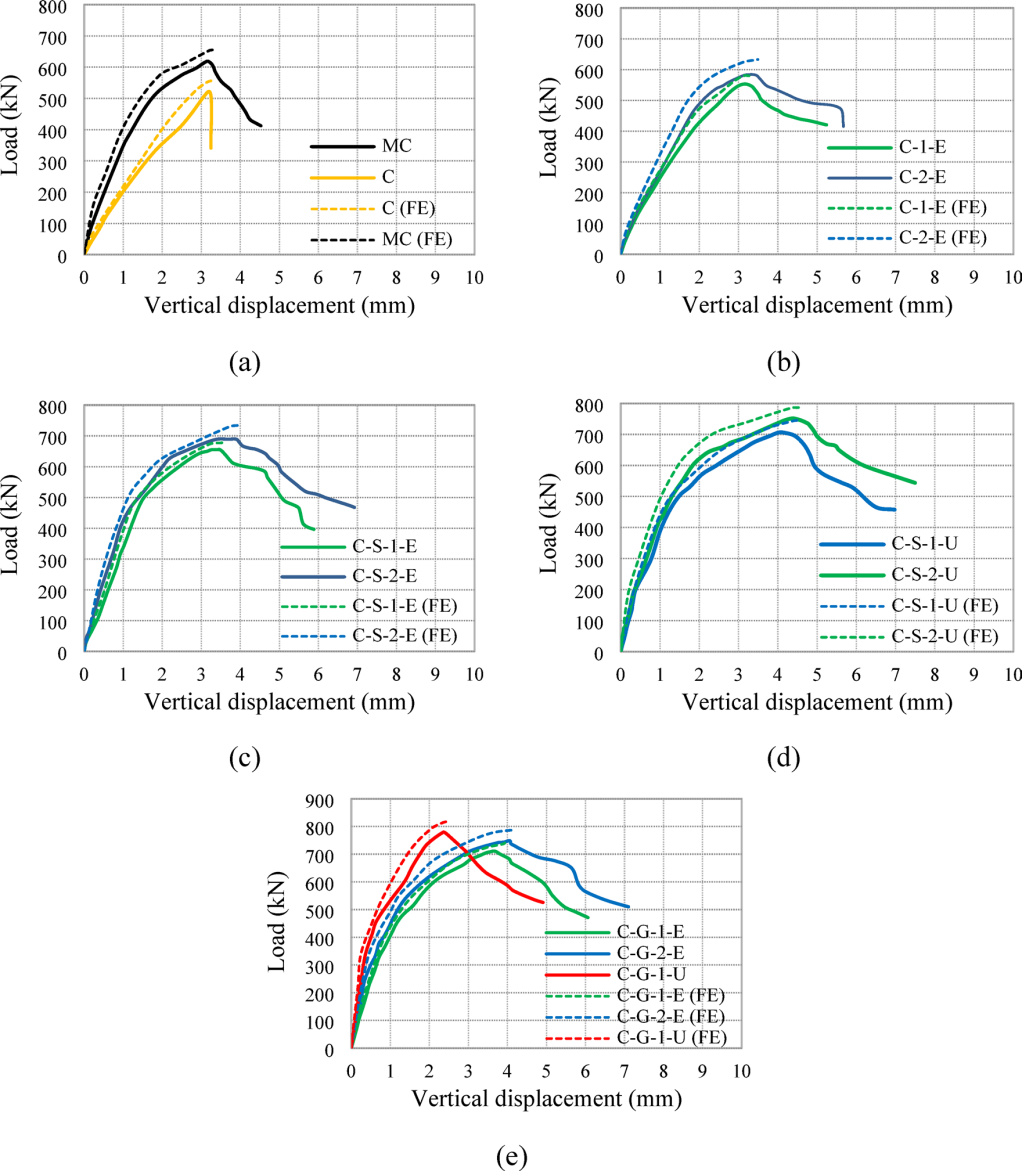
Comparison between experimental and numerical load-vertical displacement relationships: (**a**) G1, (**b**) G2, (**c**) G3, (**d**) G4, and (**e**) G5.

The finite element modeling was successfully verified against the experimental results, demonstrating its ability to accurately replicate the behavior of the tested specimens, as shown in Figs. 15 and 16. According to Table 4, the ratio of experimental to numerical ultimate load ranged between 0.92 and 0.97, with an average of 0.95, demonstrating a strong correlation between the predicted and observed peak capacities. Similarly, the predicted axial displacement at peak load displayed a close match to the experimental values, with an average EXP/FE ratio of 0.94. As illustrated in Fig. 16, the model was also able to effectively capture the overall axial load–displacement response, reflecting the stiffness and post-peak behavior observed in the physical tests. Additionally, the finite element simulations provided good agreement in predicting the failure modes of the columns (Fig. 15), accurately capturing key phenomena such as cracking patterns, delamination of strengthening layers, and localized crushing of concrete. These results confirm the robustness of the developed numerical model and its reliability in simulating the structural performance of strengthened RC columns.

**Table 4:** Comparison between numerical and experimental results.

Specimens’ ID	P_u_			Δ_Pu_		
	EXP (kN)	FE (kN)	EXP/FE	EXP (mm)	FE (mm)	EXP/FE
MC	618	655	0.94	3.14	3.28	0.96
C	522	558	0.94	3.19	3.30	0.97
C-1-E	553	578	0.96	3.16	3.37	0.94
C-2-E	583	632	0.92	3.24	3.49	0.93
C-S-1-E	655	677	0.97	3.48	3.54	0.98
C-S-2-E	689	733	0.94	3.41	3.98	0.86
C-S-1-U	705	745	0.95	4.15	4.49	0.92
C-S-2-U	751	788	0.95	4.36	4.59	0.95
C-G-1-E	711	740	0.96	3.64	3.93	0.93
C-G-2-E	748	788	0.95	4.06	4.17	0.97
C-G-1-U	779	818	0.95	2.35	2.49	0.94
Avg			0.95			0.94
SD			0.012500556			0.03406548
CoV			0.001250056			0.00340655

EX: Experiment; FE: Finite element model; Avg: Average; SD: Standard deviation; CoV: Coefficient of variation.

### Parametric study

**Fig. 17:**
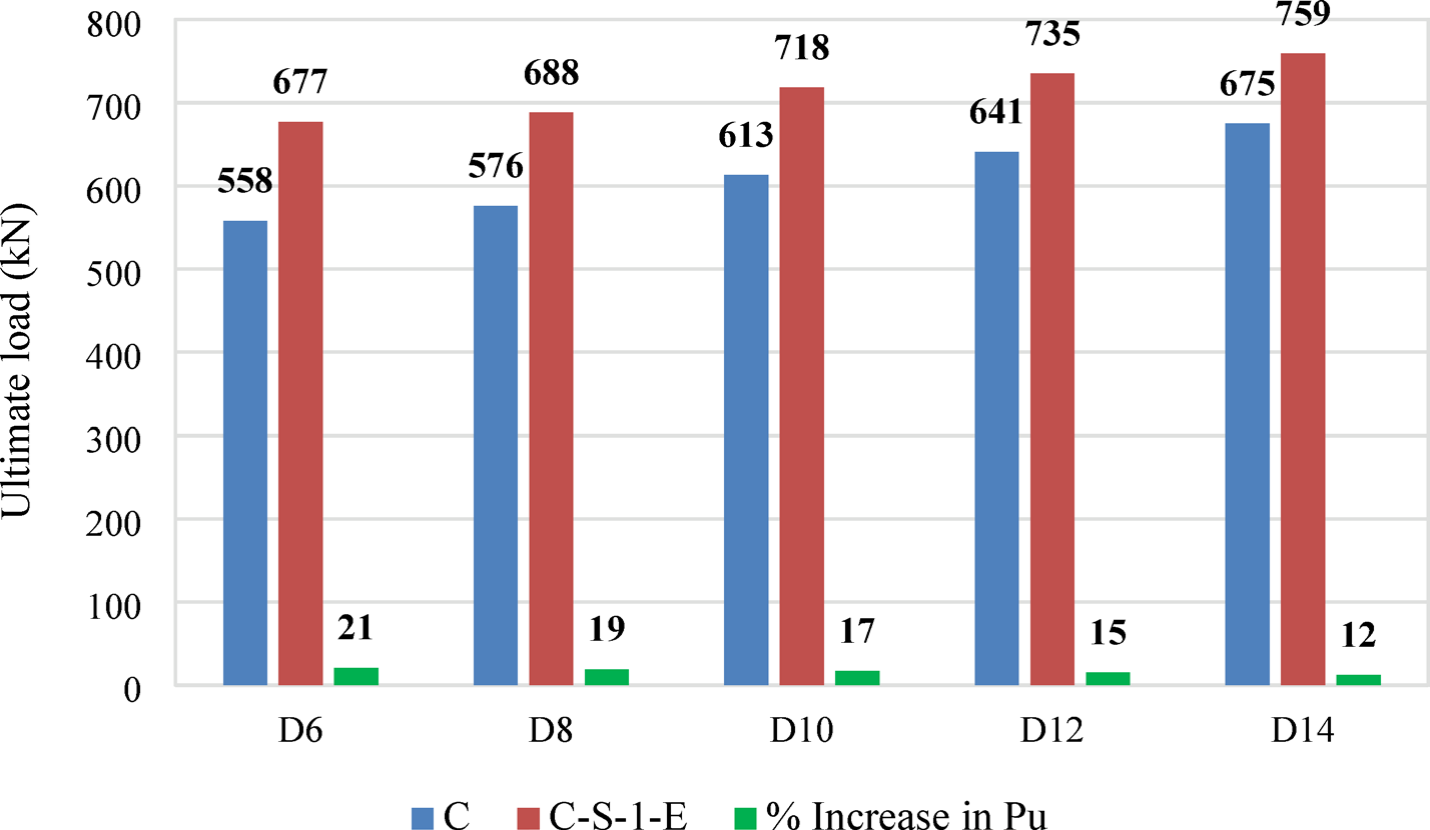
Ultimate loads of columns C-S-1-E for different bar diameters before and after strengthening.

To further evaluate the effectiveness of the proposed strengthening approach, a parametric study was conducted using specimen C-S-1-E, which was strengthened with four embedded NSM steel bars and an ECC jacket reinforced with one layer of GFRP mesh. The study examined the effect of the main reinforcement diameter. The influence of the main reinforcement diameter was analyzed by considering bar sizes of 6, 8, 10, 12, and 14 mm, while keeping other parameters constant. The obtained ultimate load capacities are presented in Fig. 17 and compared with the control deficient specimen (C), which contained 6 mm bars and failed at 558 kN.

Increasing the bar diameter from 6 mm to 8 mm resulted in a 3% increase in axial load capacity, reaching 576 kN. Using 10 mm bars further increased the ultimate load to 613 kN, representing a 9.8% enhancement over the control column. With 12 mm reinforcement, the load capacity rose to 641 kN, marking a 14.8% improvement compared with the control column. The highest load capacity of 675 kN was achieved with 14 mm reinforcement, corresponding to a 20.9% increase over the control specimen and a 12% improvement over the strengthened specimen with 6 mm bars.

These results demonstrate a clear positive correlation between bar diameter and axial load capacity, where increasing steel reinforcement enhanced load resistance and delayed failure. However, it is worth noting that the rate of strength increase diminishes for larger diameters, indicating the influence of concrete confinement limits.

## Conclusions

This study explores a novel strengthening method using NSM steel or GFRP bars with an external ECC or UHPC layer reinforced with GFRP mesh. Key parameters include the type of strengthening layer, the presence and number of GFRP mesh layers (1 or 2), and the combined effect of NSM bars with external jacketing. Based on the results, the following conclusions are drawn:

- The observed failure modes highlight that NSM steel bars effectively redistributes stress and delays brittle failure. Columns strengthened with NSM steel bars and ECC/UHPC jackets exhibited improved ductility, with two-layer GFRP mesh configurations providing superior confinement compared with single-layer systems.
- Replacing NSM steel bars with NSM GFRP bars resulted in distinct failure patterns, with ECC-confined columns showing a more ductile response and UHPC-confined specimens exhibiting localized rupture due to their increased stiffness.
- The combination of NSM reinforcement with external confinement significantly enhances the resilience of deficient columns under axial loading.
- Strengthening with ECC and GFRP mesh led to moderate improvements, increasing the ultimate load by 6%–12% while reducing failure brittleness.
- Incorporating NSM steel bars with ECC notably enhanced performance, increasing ultimate load capacity by 25%–32%, with energy absorption by up to 3.9 times compared with the control column.
- Using NSM steel bars with UHPC further improved confinement, achieving 35%–44% higher loads and energy absorption up to 4.70 times that of the control column.
- The best performance was achieved using NSM GFRP bars with UHPC, with a maximum load increase of 49%, indicating superior structural resilience.
- Columns with larger reinforcement diameters exhibited higher load capacities; however, the rate of improvement decreased with increasing bar size, suggesting that optimal reinforcement ratios should be considered for maximum efficiency.

## Data Availability

Data will be made available on request.
